# The LAMMER Kinase, LkhA, Affects *Aspergillus fumigatus* Pathogenicity by Modulating Reproduction and Biosynthesis of Cell Wall PAMPs

**DOI:** 10.3389/fcimb.2021.756206

**Published:** 2021-10-13

**Authors:** Joo-Yeon Lim, Yeon Ju Kim, Seul Ah Woo, Jae Wan Jeong, Yu-Ri Lee, Cheol-Hee Kim, Hee-Moon Park

**Affiliations:** ^1^ Laboratory of Cellular Differentiation, Department of Microbiology and Molecular Biology, College of Bioscience and Biotechnology, Chungnam National University, Daejeon, South Korea; ^2^ Institute of Biotechnology, Chungnam National University, Daejeon, South Korea; ^3^ Laboratory of Developmental Genetics, Department of Biology, College of Bioscience and Biotechnology, Chungnam National University, Daejeon, South Korea

**Keywords:** *Aspergillus fumigatus*, gene regulation, fungal development, interactions with host cells, molecular mechanisms of fungal pathogenesis, pathogen associated molecular patterns (PAMPs)

## Abstract

The LAMMER kinase in eukaryotes is a well-conserved dual-specificity kinase. *Aspergillus* species cause a wide spectrum of diseases called aspergillosis in humans, depending on the underlying immune status of the host, such as allergy, aspergilloma, and invasive aspergillosis. *Aspergillus fumigatus* is the most common opportunistic fungal pathogen that causes invasive aspergillosis. Although LAMMER kinase has various functions in morphology, development, and cell cycle regulation in yeast and filamentous fungi, its function in *A. fumigatus* is not known. We performed molecular studies on the function of the *A. fumigatus* LAMMER kinase, *Af*LkhA, and reported its involvement in multiple cellular processes, including development and virulence. Deletion of *AflkhA* resulted in defects in colonial growth, production of conidia, and sexual development. Transcription and genetic analyses indicated that *Af*LkhA modulates the expression of key developmental regulatory genes. The *AflkhA*-deletion strain showed increased production of gliotoxins and protease activity. When conidia were challenged with alveolar macrophages, enodocytosis of conidia by macrophages was increased in the *AflkhA*-deletion strain, resulting from changes in expression of the cell wall genes and thus content of cell wall pathogen-associated molecular patterns, including β-1,3-glucan and GM. While T cell-deficient zebrafish larvae were significantly susceptible to wild-type *A. fumigatus* infection, *AflkhA*-deletion conidia infection reduced host mortality. *A. fumigatus Af*LkhA is required for the establishment of virulence factors, including conidial production, mycotoxin synthesis, protease activity, and interaction with macrophages, which ultimately affect pathogenicity at the organismal level.

## Introduction

*Aspergillus fumigatus* is an opportunistic fungal pathogen that causes aspergillosis in immunocompromised individuals. Human infections caused by *Aspergillus* can range from allergenic disease to severe invasive aspergillosis (IA). *A. fumigatus* can grow and reproduce in soil, air dust, and decompose organic matter as a saprotroph. Conidia (2–3 μm), which are asexual spores, are sufficient to bypass mucociliary clearance and reach the alveoli in the lungs (Abad et al., 2010). While clearance of inhaled conidia occurs as a result of a highly coordinated immune response in immunocompetent hosts, conidia can efficiently adapt their physiology to the altered host environment in immunocompromised individuals. Epithelial barriers and innate immune cells, such as neutrophils, macrophages, and dendritic cells, play important roles in the removal of fungal invaders (Osherov, 2012). To recognize a pathogen, host cells contain pattern recognition receptors (PRRs), including Toll-like receptors (TLRs), C-type lectin receptors (CLRs), and NOD-like receptors (NLRs) (Van De Veerdonk et al., 2017). PRRs interact with pathogen-associated molecular patterns (PAMPs), which are usually absent from the host as essential structural components of pathogens (Hatinguais et al., 2020). Fungal cell wall components in conidia and hyphae are characteristic PAMPs, including glucan, chitin, and galactomannan (GM) (Marcos et al., 2016). Conidia have a protective outer layer comprised of hydrophobins and melanin and shed this outer layer during germination, exposing PAMPs to PRRs (Aimanianda and Latgé, 2010; Perez-Cuesta et al., 2020). The innate immune system induced by PAMP-PRR interaction causes phagocytosis, killing of fungi, and cytokine signaling for activation of the adaptive immune response. Stimulated macrophages cause phosphorylation and activation of mitogen-activated protein kinases (MAPKs) and produce pro-inflammatory cytokines and chemokines, such as tumor necrosis factor-α (TNF‐α) and interleukins (IL-1α, IL-6, and IL-8) (Rao, 2001; Dubourdeau et al., 2006; Dagenais and Keller, 2009). TNF-α enhances the host immune response by augmenting the phagocytic potential of phagocytes and by regulating the growth and differentiation of a wide range of cells.

Fungal products may contribute to fungal pathogenicity in immunocompromised hosts by evading or modulating host defenses. Several molecules secreted by *A. fumigatus* can inhibit the phagocytic activity of the host, including galactosaminogalactan (GAG), mycotoxins, such as fumagillin and gliotoxin (GT), and hydrolytic enzymes. GAG is a specific carbohydrate polymer that consists of galactose, galactosamine, and N-acetylgalactosamine, and is expressed by hyphae and secreted (Van De Veerdonk et al., 2017). GAG plays crucial roles in the adherence of hyphae to surfaces and formation of biofilms, can induce neutrophil apoptosis and increase resistance to neutrophil extracellular traps (NETs) (Fontaine et al., 2011; Lee et al., 2015). Mycotoxin fumagillin and GT can decrease the function of neutrophils and macrophages (Fallon et al., 2010; Schlam et al., 2016). While less than 20% of the environmental isolates produced GT, approximately 93% of *A. fumigatus* strains recovered from cancer patients with IA produced GT (Lewis et al., 2005; Sugui et al., 2007). *A. fumigatus* produces a wide range of proteases and hydrolytic enzymes to catalyze macromolecules, which enables the fungus to grow and survive in distinct environments (de Vries and Visser, 2001). The *A. fumigatus* genome contains more than 100 proteases (Watson et al., 2011). Alkaline protease 1 (Alp1) is the most abundant protein secreted by *A. fumigatus* and is detected in the airways of patients with asthma, but not in healthy individuals (Balenga et al., 2015).

LAMMER kinase is a dual-specificity kinase that phosphorylates serine/threonine and tyrosine residues (Yun et al., 1994). LAMMER kinases containing a conserved motif, ‘EHLAMMERILG,’ in sub-domain X of the kinase catalytic domain, are highly conserved in eukaryotes and have multiple functions in various physiological processes: growth, cell wall biogenesis, stress response, cell division, and differentiation (Lim and Park, 2019b). In contrast to the bacterial pathogen, *A. fumigatus* undergoes morphological transition in the host environment during infection: inert conidia swelling, germination, and growth into lung tissue, until finally undergoing dissemination. Various signaling pathways are involved in the morphogenesis and development of fungi. We investigated the multifunctional role of LAMMER kinase in morphogenetic plasticity and development, which are intimately coupled with virulence in pathogenic fungi.

To investigate the cellular function of LAMMER kinase in *A. fumigatus*, we cloned *AflkhA*, which is a homolog of *Aspergillus nidulans AnlkhA*. Deletion of *AflkhA* resulted in defects in colonial growth, conidia production, sexual development, a decreased ability to form biofilms, and increased protease activity and GT production. Additionally, deletion of *AflkhA* resulted in increased susceptibility to phagocytosis by alveolar macrophages and increased survival rate in a T cell development-deficient zebrafish infection assay. Here, we present, for the first time, that LAMMER kinase is involved in the development and virulence of the fungal pathogen *A. fumigatus*.

## Results

### *Af*LkhA Is Required for Colonial Growth and Asexual Development in *A. fumigatus*

We performed BLASTP analysis of the *A. fumigatus* genome database using *A. nidulans An*LkhA as a query and identified *Af*LkhA (AFUA_1G16780) as the best hit (identity = 81%). *Af*LkhA is the only LAMMER kinase ortholog in *A. fumigatus* and contains 664 amino acids (73 kDa). Most of the LAMMER kinase orthologs in *Aspergillus*, except *Aspergillus glaucus* and *Aspergillus terreus*, include “EHLAMMEAVIG” as a LAMMER motif in the catalytic domain (**Supplementary Figure 1**). The catalytic domain of *Af*LkhA comprises 3^rd^–6^th^ exons. With a high identity, *Af*LkhA might have functions similar to those of *An*LkhA.

**Figure 1:**
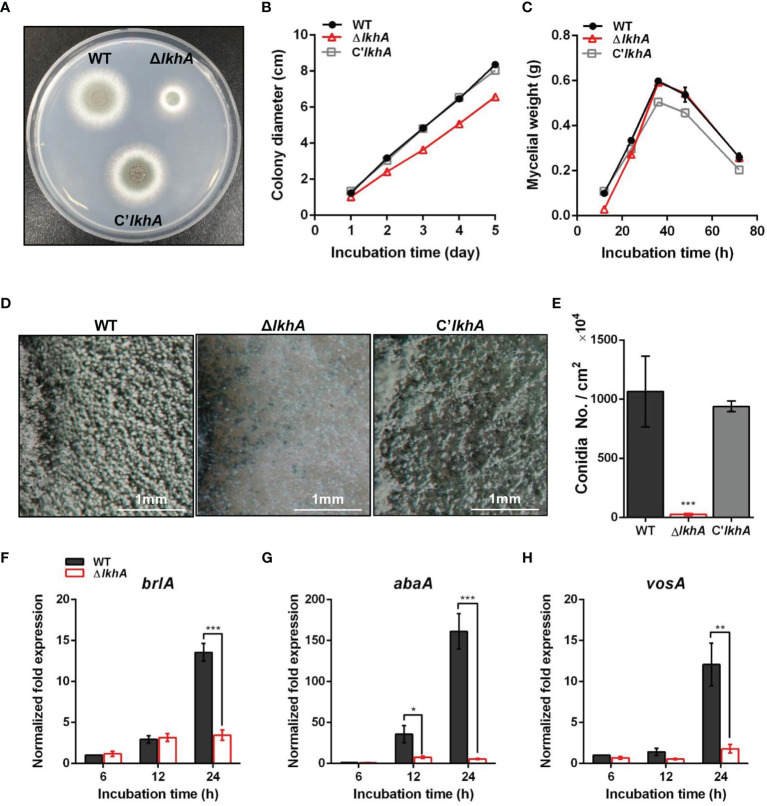
Vegetative growth and asexual development of Δ*AflkhA* strain. **(A)** Colony morphology of WT, Δ*AflkhA*, and C’*AflkhA* strains. Spores were inoculated onto GMM agar and incubated at 37°C for two days. **(B)** Radial growth of WT, Δ*AflkhA*, and C’*AflkhA* strains. Colony diameter of each strain was measured daily from point of inoculation over five days. When error bars are not shown, they are smaller than symbols (n = 5). **(C)** Mycelial production on liquid YCGMM. Dry weight of each strain was measured. When error bars are not shown, they are smaller than symbols (n = 3). **(D)** Conidiophore heads on GMM agar. **(E)** Quantification of conidia production (n = 4). *P < 0.05. **(F-H)** Expression patterns of *brlA* **(F)**, *abaA* **(G)** and *vosA* **(H)** genes. Mycelial balls of strains obtained from YCGMM liquid culture were transferred to GMM agar and incubated for indicated hrs. Total RNA was extracted and RT-qPCR analysis was performed using 18S rRNA gene as an internal control. **P < 0.01, ***P < 0.001.

To characterize the functions of *Af*LkhA, we generated the *AflkhA*-deletion (Δ*AflkhA*) strain by replacing its open reading frame (ORF) with the *ptrA* marker in the AFIR928 strain (*MAT1-2*), which is a supermater strain. The Δ*AflkhA* strain was viable but showed reduced growth on agar medium (**Figures 1A, B**). There was no remarkable difference in the dry weight of liquid cultures among the wild type (WT), Δ*AflkhA*, and C’*AflkhA* strains (**Figure 1C**).

*Af*LkhA affects both vegetative growth and asexual development. Under a stereomicroscope, there was a reduction in the formation of conidiophores on the Δ*AflkhA* colony, compared to those of the WT (**Figure 1D**). In agreement with the phenotypic data, statistical analysis revealed an approximately 98% decrease in conidiospore production in the Δ*AflkhA* strain (**Figure 1E**). BrlA, AbaA, and WetA constitute the backbone of the central regulatory pathway (CRP) and regulate asexual development in *Aspergillus* (Etxebeste et al., 2010; Yu, 2010). Mycelia balls produced in liquid YCGMM were shifted to solid GMM to induce asexual development, and total RNA was prepared from the cultures at the indicated time post-induction. When we investigated the expression levels of CRP genes *brlA* and *abaA*, the Δ*AflkhA* strain showed significantly reduced transcript levels of *brlA* at 24 hr, and *abaA* at 12 and 24 hrs (**Figures 1F, G**). VosA is a Velvet family protein, which plays an important role in spore viability (Park et al., 2012). The level of *vosA* expression in the Δ*AflkhA* strain was reduced (**Figure 1H**). Taken together, these results indicate that *AflkhA* plays a pivotal role in the production of conidiophores. To elucidate the cellular function of LAMMER kinase in stress response, spotting analysis was performed on agar containing several stress chemicals. No significant changes were observed in the sensitivity to cell wall stress (calcofluor white and Congo red), oxidative stress (H_2_O_2_ and menadione), osmotic stress (KCl and NaCl), ion depletion stress (EDTA and EGTA), and endoplasmic reticulum stress (tunicamycin and dithiothreitol) (**Supplementary Figure 2**).

### *Af*LkhA Is Required for Sexual Development

**Figure 2:**
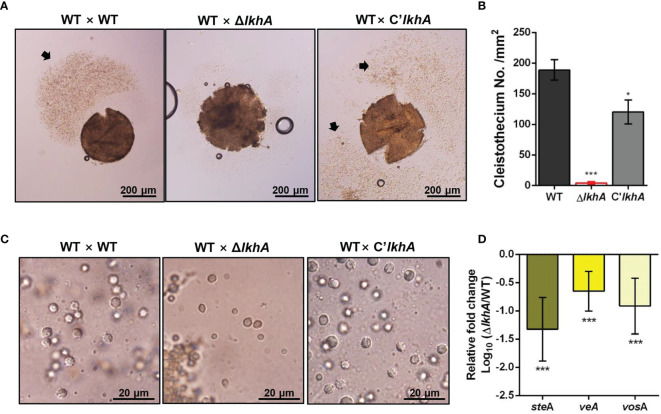
Role of *Af*LkhA in sexual development. **(A)** Morphology of the sexual fruiting body, cleistothecium, in WT, Δ*AflkhA*, and C’*AflkhA* strains. Mycelial balls were moved to oatmeal agar and incubated at 30°C for 14 days under dark conditions to induce sexual development. Arrow indicates a cloud of ascospores sprayed from cleistothecium. **(B)** Quantification of cleistothecia production (n = 5). Asterisk represents significant differences: *P < 0.05, ***P < 0.001. **(C)** Morphology of ascospores. Cleistothecium was ruptured on a glass slide to observe ascospores. **(D)** Relative expression patterns of *steA*, *veA* and *vosA*. Total RNA was extracted from cleistothecia. RT-qPCR analysis was performed using 18S rRNA gene as an internal control. The x-axis shows a list of genes. The y-axis indicate the relative mRNA abundance of the genes in Δ*AflkhA* strain compared to WT. The values were transformed and presented as Log_10_. ***P < 0.001.

LAMMER kinase is involved in both asexual and sexual development in *A. nidulans* (Kang et al., 2013). To investigate the effect of *AflkhA*-deletion on sexual development in *A. fumigatus*, crosses were induced by the VeM method on oatmeal agar under sealed and dark conditions (Lim and Park, 2019a). Crossing of AFB62 strains with AFIR928 (WT × WT) and with C’*AflkhA* (WT × C’*lkhA*) produced cleistothecia, sexual fruiting bodies, containing a cloud of ascospores (**Figure 2A**, arrow). The crossing of the WT strain with the Δ*AflkhA* strain (WT × Δ*lkhA*) produced similar sizes of cleistothecium (**Supplementary Figure 3**). The number of cleistothecia (4 ± 2 cleistothecia/mm^2^) was significantly lower than that in the WT × WT cross (189 ± 17 cleistothecia/mm^2^) and the WT × C’*lkhA* cross (120 ± 20 cleistothecia/mm^2^) (**Figure 2B**). To investigate the formation of ascospores, we picked up cleistothecia and crushed them on glass slides. While the WT × WT cross produced mature ascospores with normal equatorial crests, the WT × Δ*lkhA* cross produced abnormal ascospores with no equatorial crests (**Figure 2C**).

Next, we investigated the gene expression patterns in cleistothecia and sexual fruiting bodies. When *A. fumigatus* sexual development is induced by the VeM method, both asexual and sexual developmental reproductive organs are observed on oatmeal agar (Lim and Park, 2019a). To investigate changes in gene expression for only sexual development, we picked up ten cleistothecia from each crossing and extracted total RNAs from these sexual fruiting bodies. SteA is a transcription factor that controls filamentation and sexual development in *A. nidulans* (Vallim et al., 2000). VeA is a key developmental regulator in numerous fungal species, particularly Ascomycetes (Myung et al., 2012). VeA has been demonstrated to positively regulate sexual development in various *Aspergillus* species, including *A. nidulans*, *Aspergillus cristatus*, *Aspergillus parasiticus*, and *Aspergillus flavus* (Kim et al., 2002; Calvo et al., 2004; Duran et al., 2006; Liu et al., 2018a). VosA is required for the integrity of both asexual and sexual spores. Levels of both gene expression and proteins levels of *vosA* are high in conidia and ascospores (Ni and Yu, 2007). The expression of *steA*, *veA*, and *vosA* genes were downregulated in the WT × Δ*lkhA* cross (**Figure 2D**). Taken together, our data suggests that *Af*LkhA plays an essential role in sexual development.

### *Af*LkhA Is Required for Negative Regulation of GT

**Figure 3:**
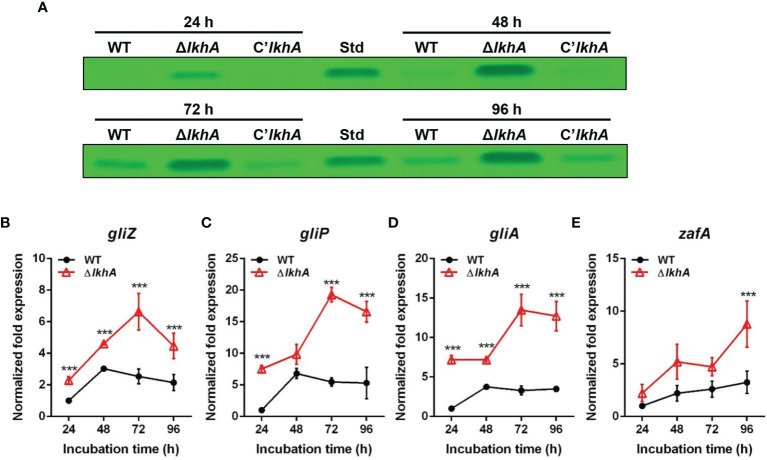
Role of *Af*LkhA in gliotoxin production. **(A)** Determination of GT production in WT, Δ*AflkhA*, and C’*AflkhA* strains. GT were extracted with chloroform from culture supernatant of each strain and subjected to TLC. Std: GT standard (Simga-Aldrich). **(B–E)** Total RNA was extracted from mycelial balls incubated in Czapek Dox broth. RT-qPCR analysis was performed using 18S rRNA gene as an internal control. Expression patterns of GT cluster genes, transcription factor *gliZ* **(B)**, nonribosomal peptide synthetase *gliP* **(C)**, transporter *gliA* **(D)**, and transcriptional activator *zafA* **(E)**. ***P < 0.001.

To test whether the absence of *Af*LkhA would affect the biosynthesis of GT, a mycotoxin considered as a putative virulence factor, the production of GT and expression of corresponding genes were investigated. Thin-layer chromatography (TLC) images showed that the Δ*AflkhA* strain produced an increased amount of GT compared to the WT and C’*AflkhA* (**Figure 3A**). GT is synthesized by biosynthetic gene clusters composed of 12 genes (Wang et al., 2014). We examined the expression of genes encoding GT biosynthetic genes (*gliP*, *gliZ*, and *gliA*). GliZ is a Zn_2_-Cys_6_ transcription factor that controls gene expression of other genes involved in GT biosynthesis (Bok et al., 2006). GliP is a nonribosomal peptide synthase that catalyzes the first step in the GT biosynthetic pathway (Sugui et al., 2007). By exporting the GT, GliA is involved in protection from extracellular GT, including its own produced toxin (Wang et al., 2014). Expression levels of *gliP*, *gliZ*, and *gliA* were significantly higher in the Δ*AflkhA* strain than those in the WT strain (**Figures 3B–D**). A zinc-responsive transcriptional activator ZafA binds to ZafA-binding motifs in the upstream region of *gliZ* (Seo et al., 2019). The expression of *zafA* was upregulated in the Δ*AflkhA* strain (**Figure 3E**). Taken together, the Δ*AflkhA* strain increases GT production by regulating expression of *zafA* gene and GT cluster genes, suggesting that *Af*LkhA negatively controls GT production.

### *Af*LkhA Is Involved in Protease Activity and Biofilm Formation

Hydrolytic enzymes are virulence factors that allow *A. fumigatus* to degrade molecules and obtain energy sources from the host environment (Richie et al., 2009). Various allergens show protease activity and cause protease-dependent release of the pro-inflammatory cytokines, IL-6, and IL-8 (Kauffman et al., 2000). We examined the possible role of *Af*LkhA in controlling protease activity in *A. fumigatus*.

**Figure 4:**
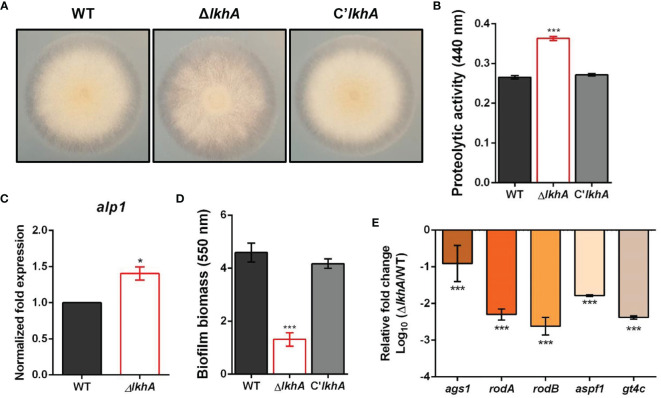
Roles of *Af*LkhA in protease production and biofilm formation. **(A)** Halo formed at edge of colonies of WT, Δ*AflkhA*, and C’*AflkhA* strains. Each strains were point-inoculated on Czapek Dox medium containing 1% skim milk and incubated at 30°C. **(B)** Quantification of proteolytic activity. Supernatants were obtained from Czapek Dox broth (pH 7.3) containing 1% skim milk powder for 3 days at 30°C. Protein concentrations were calculated with a BCA Protein Assay Kit. Protease activity was measured using an azocasein assay. The experiments included six replicates (n = 6). ***P < 0.001. **(C)** Expression pattern of alkaline protease *alp1* gene. Total RNA was extracted from mycelial balls incubated in Czapek Dox broth. RT-qPCR analysis was performed using 18S rRNA gene as an internal control. *P < 0.05. **(D)** Crystal violet staining assay. Biofilm formation was measured after 16 hrs of growth in GMM. The biofilms were stained with 0.01% crystal violet and dissolved in 30% acetic acid solution. The amounts of dye were measured by spectrophotometry at 550 nm (n = 10). ***P < 0.001. **(E)** Relative expression patterns of α-1,3-glucan synthase (*ags1*), hydrophobins *rodA*, *rodB*, a ribonuclease (*aspf1*), and a GAG synthase (*gt4c*). Total RNA was extracted from biofilm cultures. RT-qPCR analysis was performed using 18S rRNA gene as an internal control. The x-axis shows a list of genes. The y-axis indicate the relative mRNA abundance of the genes in Δ*AflkhA* strain compared to WT. The values were transformed and presented as Log_10_. ***P < 0.001.

When the cultures were grown on 1% skim milk agar, an increase in the proteolytic activity of the Δ*AflkhA* strain was visualized by degradation halos at the edge of the colonies (**Figure 4A**). Quantitative analysis using azocasein as a substrate detected a 37% increase in alkaline protease activity in the Δ*AflkhA* cultures with respect to the WT and C’*AflkhA* strains (**Figure 4B**). *A. fumigatus*-associated serine protease, Alp1, is the most powerful alkaline protease essential for both carbon and nitrogen acquisition (Sriranganadane et al., 2010). When the expression of the *alp1* gene was determined using reverse transcriptase quantitative polymerase chain reaction (RT-qPCR) at 72 hrs, the expression level of *alp1* was higher in the Δ*AflkhA* strain than that in the WT strain (**Figure 4C**). These results suggest that *Af*LkhA is involved in alkaline protease activity by affecting transcriptional regulation.

Another important feature of fungi is their ability to form biofilms, which are microbial communities attached to the surface of organic or inorganic matters. The extracellular matrix (ECM) functions in the linkage of fungal cells with themselves or the substratum during biofilm growth (Gibbons et al., 2012). Biofilm formation was determined using crystal violet, which stained the total biofilm biomass. Biofilm formation by the Δ*AflkhA* strain was reduced by 70% compared to that of the WT and C’*lkhA* strains (**Figure 4D**). The ECM in *Aspergillus* biofilm is composed of polysaccharides, pigments, and proteins, and its formation is affected by the activity of α-1,3-glucan synthases (Ags1) and hydrophobins (RodA and RodB) (Beauvais et al., 2005; Beauvais et al., 2007; Valsecchi et al., 2018). Under biofilm conditions, *rodB* genes are highly expressed, followed by *rodA*, among the seven genes for hydrophobins (RodA–RodG) (Beauvais and Latgé, 2015). Polysaccharide GAG has a role in the adherence of hyphae to surfaces, affecting biofilm formation (Fontaine et al., 2011). The *gt4c* gene, which encodes GAG synthase, is essential for GAG production (Briard et al., 2020). Ribonuclease Aspf1 is a secreted protein that is an antigen in the biofilm of *A. fumigatus* (Gastebois et al., 2013). While α-1,3-glucans and GAGs were detected in both *in vivo* and *in vitro* biofilms, the *rodA*, *rodB*, and *aspf1* genes were detected only in the ECM *in vitro* biofilm condition (Beauvais and Latgé, 2015; Liu et al., 2018b). When the expression patterns of these genes in static biofilm conditions were analyzed by RT-qPCR analysis, the Δ*AflkhA* strain showed significantly decreased expression of *ags1*, *rodA*, *rodB*, *aspf1*, and *gt4c* (**Figure 4E**). These results suggest that LkhA plays a role in biofilm formation by regulating the transcription of genes that encode ECM and secreted proteins that are involved in adherence.

### The Conidia of Δ*AflkhA* Were Susceptible to Phagocytosis by Alveolar Macrophages

**Figure 5:**
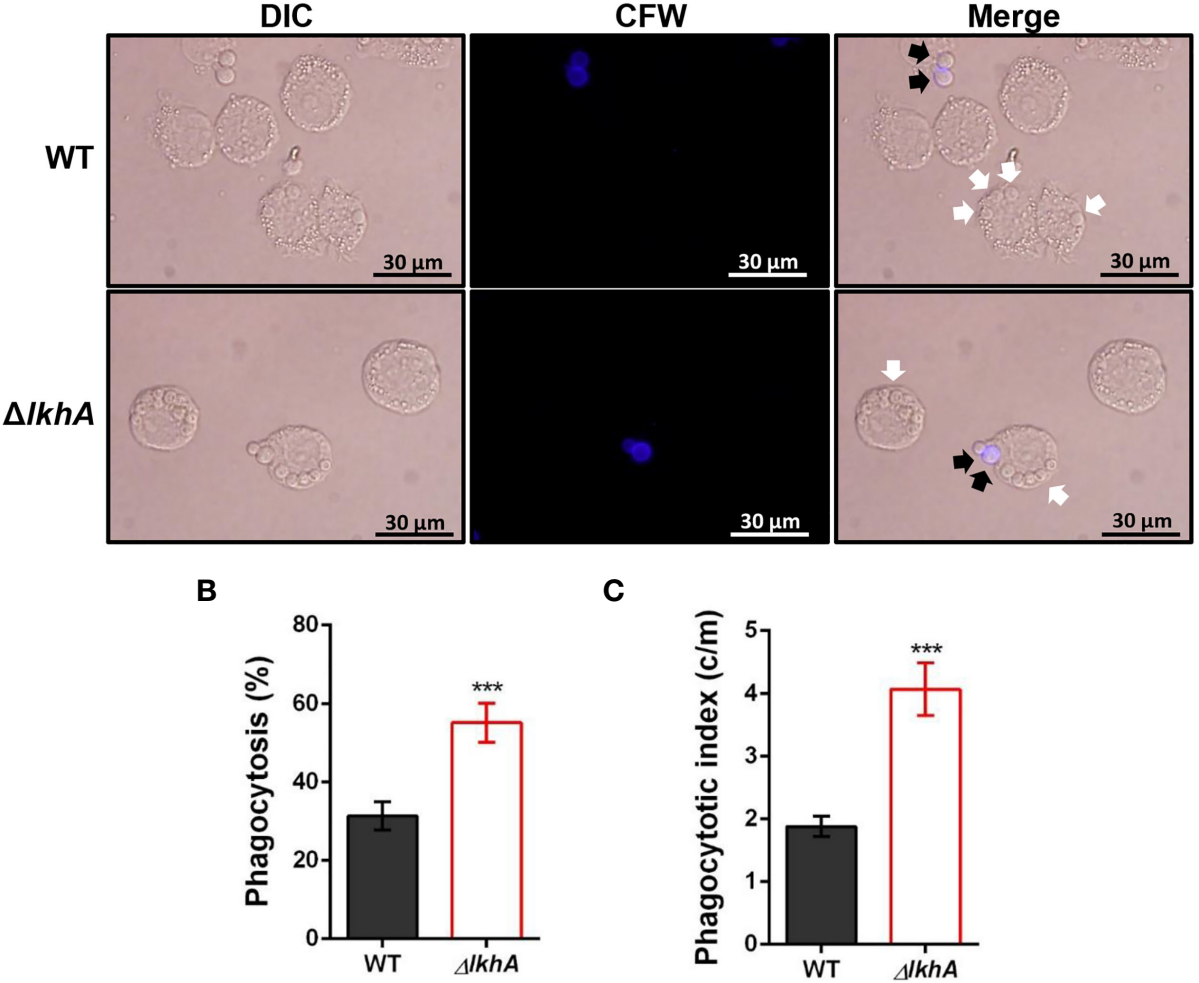
Alveolar macrophage response to Δ*AflkhA* conidia. MH-S murine alveolar macrophages were challenged with a three-fold concentration of WT and Δ*AflkhA* conidia and then incubated for 4 hrs at 37°C in an atmosphere of 5% CO_2_. **(A)** Microscopic analysis of the uptake of the fungal conidia by MH-S macrophages. External conidia (black arrows) were stained by calcofluor white (1 μg/mL in DPBS). White arrows indicate conidia endocytosed by macrophage cells. **(B)** Phagocytosis of conidia. The percentage of macrophages containing more than one ingested conidia was counted (n = 18). **(C)** Phagocytosis index. The average number of indigested conidia per macrophage (c/m) was calculated (n = 40). Statistical analysis was performed using the student’s t-test. ***P < 0.001.

To investigate whether *Af*LkhA is involved in the phagocytic process, macrophages were incubated with WT and Δ*AflkhA* conidia. Δ*AflkhA* conidia were more susceptible to phagocytosis than WT conidia (**Figure 5A**). More conidia of the Δ*AflkhA* strain (55%) were endocytosed by macrophages than those of the WT strain (31%) (**Figure 5B**). The phagocytic index (the average number of conidia per macrophage) was also greater for macrophages containing Δ*AflkhA* conidia (4.1 vs 1.9 c/m) (**Figure 5C**). Macrophages prefer to ingest Δ*AflkhA* conidia, suggesting that *Af*LkhA causes a possible change in the cell wall components of the conidia surface, which affects cell wall PAMP-PRR interactions between *A. fumigatus* conidia and host immune cells.

### *Af*LkhA Affects Content of Cell Wall PAMPs

**Figure 6:**
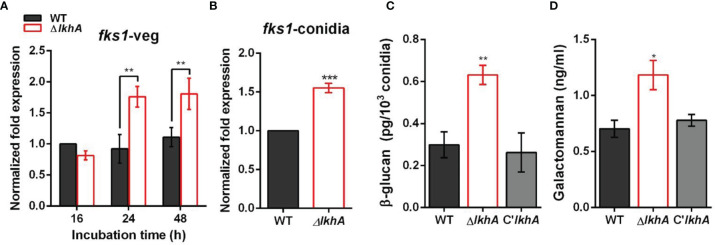
Role of *AflkhA* in biosynthesis of conidial cell walls. Expression level of β-1,3-glucan synthase *fks1* in vegetative cells **(A)** and conidia **(B)**. Spores were inoculated in MM liquid culture and incubated for the indicated number of hrs. Total RNA was extracted and RT-qPCR analysis was performed using 18S rRNA gene as an internal control. **P < 0.01, ***P < 0.001. **(C)** Amount of β-glucan (pg) per 10^3^ conidia in WT and Δ*AflkhA* strains (n = 4). **P < 0.01 **(D)** Concentration (ng/mL) of GM. Conidia was inoculated in modified Brian medium and cultured at 37°C for 24 hrs. Extracellular GM content in the culture supernatant was assayed by ELISA (n = 4). *P < 0.05.

Immune cells detect PAMPs, which are conserved molecular signatures found on the cell surface of many pathogens. To investigate the role of LAMMER kinase in the formation of the conidial cell wall, the synthesis of β-1,3-glucan was investigated. β-1,3-glucan, one of the major fungal cell wall PAMPs, is detected by Dectin-1, a C-type lectin receptor of monocytes and macrophages (Faro-Trindade et al., 2012). In *A. fumigatus*, the expression of *fks1* (encoding β-1,3-glucan synthase) was detected in swollen conidia and was detected during mycelial growth (Gastebois et al., 2013). While the *fks1* gene was consistently expressed during vegetative growth in WT, *fks1* mRNA was upregulated in the Δ*AflkhA* strain (**Figure 6A**). Increased expression of *fks1* gene was also detected in the Δ*AflkhA* conidia (**Figure 6B**). In accordance with the increased expression of the *fks1* gene, the amount of β-1,3-glucan in the Δ*AflkhA* conidia was significantly higher than that observed in the WT and C’*AflkhA* conidia (~2-fold higher in the Δ*AflkhA* strain compared to the WT) (**Figure 6C**). Next, we investigated the expression of *ags1* (encoding α-1,3-glucan synthase) and *chsG* (encoding chitin synthase), which are required for fungal cell wall synthesis (Mellado et al., 1996; Beauvais et al., 2013). The Δ*AflkhA* strain expressed increased levels of *ags1* and *chsG* (**Supplementary Figure 4**).

Next, we investigated the abundance of GM in vegetative cell cultures grown in Brian’s medium by an enzyme-linked immunosorbent assay (ELISA). GM mediates the binding of *A. fumigatus* conidia to DC-SIGN, a type II membrane C-type lectin in lung dendritic cells (DCs) and alveolar macrophages (Serrano-Gómez et al., 2004). We found that the Δ*AflkhA* strain produced more GM than the WT and C’*AflkhA* strains (~1.7 fold increase in Δ*AflkhA* compared to WT) (**Figure 6D**). Collectively, these data suggest that *AflkhA* affects fungal cell wall content, which leads to alterations in the innate immune recognition of PAMPs.

### Macrophages Challenged With Δ*AflkhA* Conidia Induced Early ERK Activation and Produced More Cytokine TNF- α

**Figure 7:**
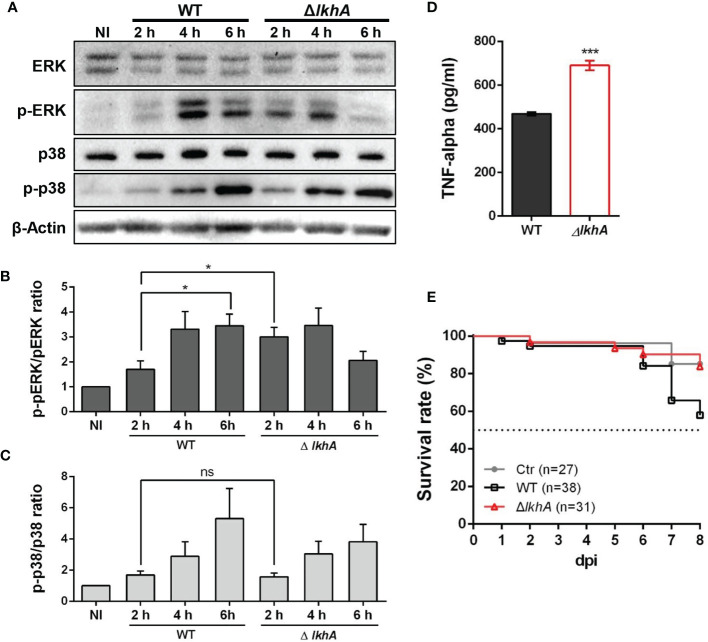
Effect of MAPK phosphorylation and cytokine release from alveolar macrophages treated with *AflkhA*-null conidia. MH-S cells were simulated with 10-fold of WT and Δ*AflkhA* conidia and then incubated for the indicated time points. **(A)** Phosphorylation of ERK and p38 after stimulation of MH-S cells with conidia was analyzed by western blotting. The level of phosphorylation was quantified after normalization to the levels of total ERK **(B)** or p38 **(C)** using ImageJ software. *P < 0.05. ns, not significant. **(D)** Release of TNF-α. MH-S cells were challenged with 10-fold WT and Δ*AflkhA* conidia. The release of TNF-α was measured by ELISA 6 hrs after infection. ***P < 0.001. **(E)** Virulence of *A. fumigatus* Δ*AflkhA* in T cell-deficient zebrafish larvae (*foxn1* morphant). Conidia diluted in fluorescence dye were injected at 3 dpf (0 dpi). Survival was monitored at > 8 dpi. Ctr represents the control. Data are mean values.

Following pathogen recognition, the function of alveolar macrophages is dependent in part on MAPKs phosphorylation. Extracellular signal-regulated kinase (ERK) and p38 are MAPKs that are activated and subsequently translocate to the nucleus for transcriptional activation of various target genes in macrophage cells (Rao, 2001). We studied the activation of ERK and p38 in MH-S cells after infection with WT and Δ*AflkhA* conidia (**Figure 7A**). When macrophages were stimulated with WT conidia, ERK and p38 were strongly activated, as they displayed a significant mean fold of phosphorylation of ~3.5 for ERK (**Figure 7B**, WT) and ~4.1 for p38 (**Figure 7C**, WT) after 6 hrs as compared with non-infected cells. We found a strong response of ERK (phosphorylation fold of 3.0) at an early time point (2 hrs) after infection with Δ*AflkhA* conidia (**Figure 7B**, Δ*lkhA*), but no significant difference in phosphorylation patterns of p38 compared to cells challenged with WT conidia (**Figure 7C**, Δ*lkhA*).

The proinflammatory cytokine TNF-α is produced in response to *A. fumigatus* conidia both *in vivo* and *in vitro* (Hohl et al., 2005; Dubey et al., 2014). The production of TNF-α was assessed in alveolar macrophage cells co-cultured with conidia of WT and Δ*AflkhA* strains for 6 hrs. Cells challenged with Δ*AflkhA* conidia contained substantially more TNF-α (690 ± 21) than cells with WT conidia (469 ± 8) (**Figure 7D**).

### A Lack of *Af*LkhA Attenuates Virulence of *A. fumigatus* in the T Cell-Deficient Zebrafish Larvae Model

Our results showed that macrophages responded differently to WT and Δ*AflkhA* conidia (**Figure 5**). A previous report showed that the lack of an adaptive immune system in a T cell-deficient zebrafish model is suitable for studying the role of the innate immune system in pathogenicity (Rosowski et al., 2018a). To investigate the effects of innate immunity against WT and Δ*AflkhA* conidia in an *in vivo* model system, virulence tests were performed using T cell-deficient zebrafish larvae (*foxn1* morphants) as an immunocompromised model. Forkhead box protein N1 (*foxn1*) encodes a wing-helix forkhead transcription factor that is required for thymic epithelial cell development (Lv et al., 2020). Nude mice with *Foxn1* mutations have been used as xenograft models (Fogh et al., 1977). T cell-deficient larvae were highly susceptible to WT conidia infection, with ~42% mortality at 8 days post-infection (dpi), whereas the Δ*AflkhA* conidia abrogated the effects on mortality by ~16% (**Figure 7E**). Control mock infection resulted in a mortality rate of ~15%. These data suggest that *Af*LkhA contributes to fungal pathogenicity.

## Discussion

LAMMER kinase plays a diverse role in both the development and stress responses of fungi (Lim and Park, 2019b). In this study, we performed BLASTP analysis using the *An*LkhA sequence, identified one LAMMER kinase ortholog, constructed a deletion mutant, and characterized its function in the opportunistic fungal pathogen *A. fumigatus*.

The Δ*AflkhA* strain showed defects in colonial growth, and in both asexual and sexual development. The reduced radial growth and sporulation in the Δ*AflkhA* strain agreed with previous findings in *A. nidulans*. The *lkhA*-deletion strain in *A. nidulans* showed defects in conidiophore morphology and in sporogenesis during asexual development (Kang et al., 2013). The infectious life cycle of *A. fumigatus* begins with the production of conidia, which are easily spread in the air. Reproducing asexually allows the pathogen to proliferate in the host environment. *Af*LkhA, which plays a role in conidia production, may affect the dispersal and invasion of *A. fumigatus* during inhalation and infection in the host.

*An*LkhA affects the maturation of sexual reproductive organs (cleistothecia), regulating the COP9 signalosome (CSN) *csnD* and a psi factor *ppoA* genes (Kang et al., 2013). The cell cycle and sexual differentiation of fungi, such as the fission yeast *Schizosaccharomyces pombe* and the plant pathogen *Ustilago maydis*, are affected by the deletion of LAMMER kinase (Yu et al., 2013; de Sena-Tomás et al., 2015). In addition, the LAMMER kinases of higher eukaryotes (tobacco PK12 and *Drosophila* DOA) modulate development and gene expression (Yun et al., 1994; Savaldi-Goldstein, 2003). Mutations in DOA cause defects in various structures irrespective of sex, and is required for sex determination (Du et al., 1998). As shown in **Figure 2**, the cross with the Δ*AflkhA* strain produced a decreased number of cleistothecia with abnormal ascospores due to the decreased transcription of sexual genes, *steA*, *veA*, and *vosA*. These data suggest that *AflkhA* is a novel regulator required for the completion of sexual development in *A. fumigatus*. The ability to reproduce sexually allows fungal pathogens to adapt to hostile environments; therefore, the sexual cycle can influence virulence in some pathogenic fungi (Butler, 2010). In *Crytococcus neoformans*, the basidiospores are infectious propagules in capsules that protect fungi from various stresses (Botts et al., 2009). *Aspergillus* ascospores are resistant to environmental stresses, including azole drugs (Zhang et al., 2017), suggesting that sexual reproduction is involved in the generation of resistant spores. Therefore, the contribution of LkhA to both asexual and sexual development may affect the pathogenicity of the opportunistic human pathogen *A. fumigatus* in nature.

Mycotoxins and hydrolytic enzymes are associated with virulence in *A. fumigatus*. Among mycotoxins, GT has attracted the most attention and proven activity. GT exerts a broad spectrum of immunosuppressive effects and is present in the sera of patients with IA. One possible mechanism of GT is the suppressive effect on host immune cells: inhibition of cytokine production, apoptosis of immune cells, and reduced activity of cytotoxic T-cells (Kupfahl et al., 2006; Arias et al., 2018). GT production is regulated by ZafA, the activation of which is dependent on the availability of zinc. The Δ*AflkhA* strain produced increased amounts of GT and showed increased transcription of GT cluster genes and *zafA* gene compared to WT (**Figure 3**), suggesting that LkhA negatively regulates GT production by controlling transcription of GT cluster genes and *zafA* gene in *A. fumigatus*.

*A. fumigatus* lives as a saprotroph in the environment, and most degradative enzymes are specific to plant cell wall components. However, as an opportunistic pathogen, this fungus possesses various enzymes required for infection of human tissues. In this study, we showed increased alkaline protease activity in the Δ*AflkhA* strain (**Figures 4A–C**). Based on the fact that the Δ*AflkhA* strain showed increased GT production and protease activity, we predicted an increase in pathogenicity in the Δ*AflkhA* strain. Surprisingly, the virulence of the Δ*AflkhA* strain was reduced in the T cell-deficient zebrafish larva model (**Figure 7E**). It is noteworthy that GT is not an essential virulence factor in immunosuppressed mice (Kupfahl et al., 2008).

Cell adhesion and biofilm formation are related to cell wall components. Although no significant change was observed in the sensitivity test for cell wall damaging agents (**Supplementary Figure 2A**), biofilm formation was significantly reduced in the Δ*AflkhA* strain (Fig 4D). Deletion of *AflkhA* reduced the expression of genes (*ags1*, *rodA*, *rodB*, *aspf1*, and *gt4c*) that are highly expressed in biofilms (**Figure 4E**). The ECM of biofilms *in vivo* contains α-1,3-glucans, GM, and GAGs (Beauvais and Latgé, 2015). In *A. fumigatus*, α-1,3-glucans polysaccharides are synthesized by three α-1,3-glucan synthases: Ags1, Ags2, and Ags3. Only the *ags1*-deletion strain showed a reduction in α-1,3-glucan content (Beauvais et al., 2005). During conidia swelling, cell wall α-1,3-glucans are exposed at the cell surface to function as an adhesin for the induction of aggregation (Fontaine et al., 2010). GAG is present on the surface of hyphae and covers up β-1,3-glucans from recognition by Dectin-1, which leads to decreased pulmonary inflammation (Gravelat et al., 2013). The Δ*uge3* and Δ*gt4c A. fumigatus* strains lacking UDP-glucose-4-epimerase and GAG synthase, respectively, cannot produce GAG and show decreased adherence capability and biofilm formation, suggesting that GAG acts as a major adhesin (Gravelat et al., 2013; Briard et al., 2020). It is also noteworthy that the involvement of LAMMER kinase in cell wall integrity has been reported in *Candida albicans* and *A. nidulans* (Choi et al., 2014; Lim et al., 2020b). These results suggest that *Af*LkhA affects the cell wall composition of hyphae, resulting in a decrease in biofilm formation.

The Δ*AflkhA* conidia were phagocytosed more than those of the WT by alveolar macrophage cells *in vitro* (**Figures 5A–C**). Although one of the important step in pathogenesis is germination, how germination affects immune activation and fungal clearance is unknown. Recently, it is reported that a fast-germinating strain is cleared better than a slow-germinating strain in zebrafish larvae model (Rosowski et al., 2018b). The conidia from both the WT and the Δ*AflkhA* revealed initiation of germination in 3 hrs, but the Δ*AflkhA* showed reduced germination rate thereafter than the WT (**Supplementary Figure 5**). Next, we hypothesized that cell wall PAMPs of the Δ*AflkhA* conidia differed in composition from those in WT. An increased amount of β-1,3-glucan and GM was detected in the Δ*AflkhA* strain (**Figure 6**). β-1,3-glucan and GM are recognized by PRRs of alveolar macrophages (Marcos et al., 2016). Therefore, it is possible that increased amount of PAMPs, including β-1,3-glucan and GM in the Δ*AflkhA* strain induces binding with PRRs of alveolar macrophages, which renders the Δ*AflkhA* strain susceptible to phagocytosis.

Conidia-infected bone marrow macrophages lacking Dectin-1 showed decreased TNF release and reduced phosphorylation of ERK, suggesting that Dectin-1 is required for interaction with conidia and inflammasome activation (Briard et al., 2019). When macrophages were challenged with conidia, earlier phosphorylation of ERK and increased TNF-α secretion were induced by the Δ*AflkhA* conidia (**Figures 7A–D**). Although further investigation is required, these results demonstrate that increased β-1,3-glucan in the Δ*AflkhA* conidia activates Dectin-1-mediated signal transduction, which in turn activates the adaptive immune system of the host. Dectin-1 signaling through Syk-CARD9-MALT1 activates NF-κB and induces the expression of cytokines and chemokines, including TNF-α, IL-6, IL-1α, IL-1β, G-CSF, GM-CSF, and MIP-1 (Werner et al., 2009; Faro-Trindade et al., 2012; Briard et al., 2019).

**Figure 8:**
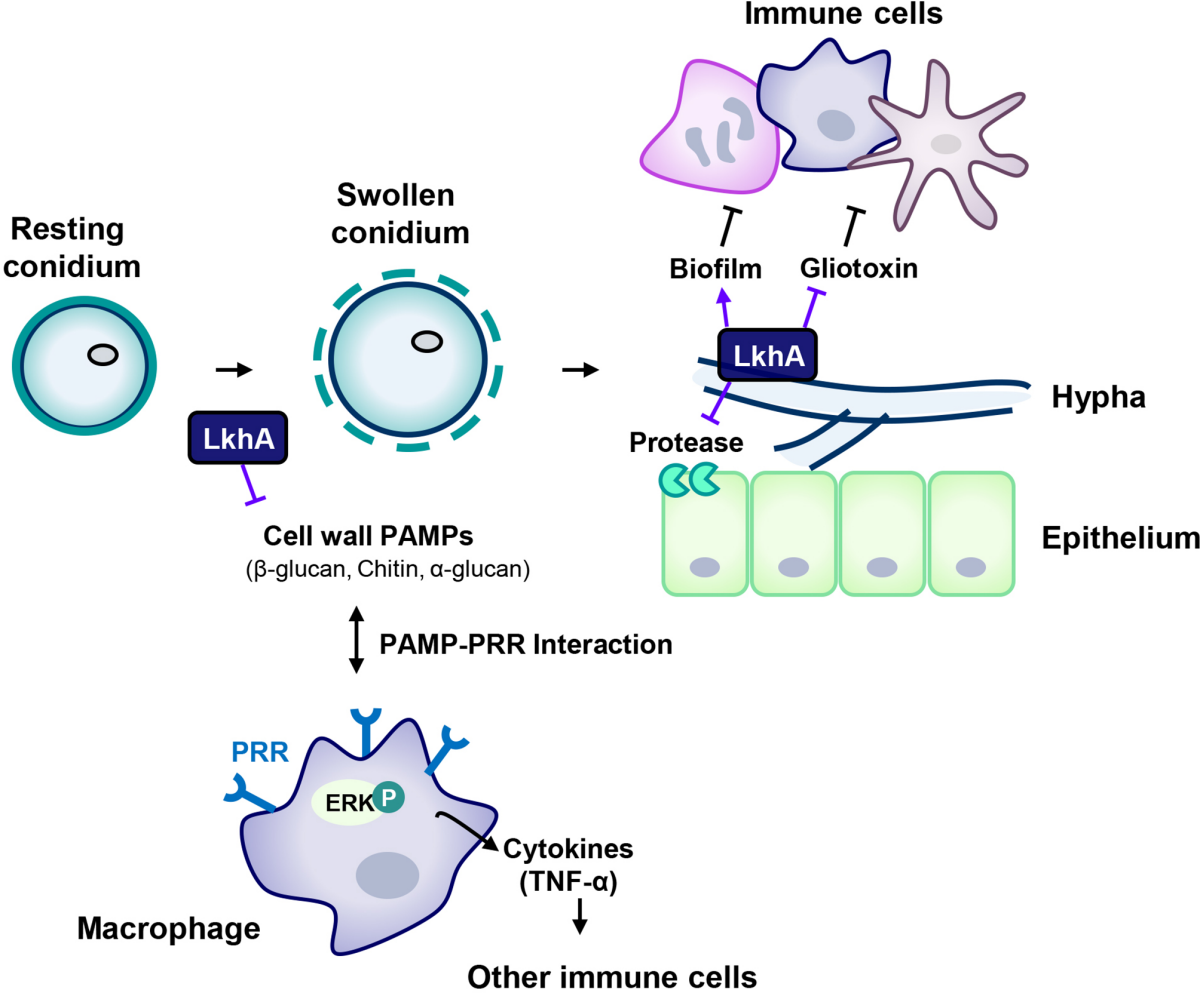
Proposed model for the involvement of LAMMER kinase in the interaction between *A. fumigatus* and immune cells. The composition of the cell wall continuously changes during the life cycle of *A. fumigatus*. The resting conidia first come into contact with host epithelial cells. When conidia swell, the outer layer (melanin and hydrophobins) is removed, uncovering the inner cell wall, which is mainly composed of glucan, chitin, and GM. LkhA regulates the transcription of genes for cell wall components and hydrophobins, which affects PAMP-PRR interactions in alveolar macrophages. This interaction mediates ERK signaling and stimulates the production of the proinflammatory cytokine TNF-α, which can promote the activation and proliferation of T cells. Hyphae differ in cell wall composition. The cell wall molecule GAG is secreted from hyphae and is present in the biofilm. The adhesion ability of *A. fumigatus* to the cell surface is important for virulence. LkhA is required for biofilm formation by modulating gene expression. During its growth, *A. fumigatus* produces GT, which is toxic to host cells. LkhA represses the expression of GT clustered genes and thus the production of GT. *A. fumigatus* also produces proteases to obtain nutrients by degrading macromolecules in the host environment. LkhA negatively regulates the expression of gene(s) for alkaline proteases and thus protease production. The virulence of *A. fumigatus* in the T cell-deficient zebrafish larva model was reduced by the loss of *lkhA*.

When in alveoli, resting conidia begin to swell, and fungal PAMPs are exposed to host immune cells after removal of the outer cell wall layers of the conidia. Following germination, mycelia grow and form biofilms, which protect microbes against hostile environments in the host immune system. Although the mycelia of Δ*AflkhA* produced increased GT and alkaline protease activity *in vitro*, the virulence of the Δ*AflkhA* strain was decreased in an *in vivo* T cell-deficient zebrafish infection model system. The reduced virulence of the Δ*AflkhA* strain could be explained by structurally or qualitatively changed PAMPs in conidia, which affects recognition by PRRs of immune cells in the innate immune response. Following the PAMPs-PRRs interaction, macrophages activate ERK to increase the secretion of the cytokine TNF-α, which mediates the transition from innate to adaptive immunity (**Figure 8**). Cytokines produced by macrophages enhance cellular immune responses to eliminate conidia by recruiting other immune cells for the release of further cytokines (Park and Mehrad, 2009). We suggest that the regulation of *Af*LkhA on the biosynthesis of fungal PAMPs may be one of the contributing factors for turning on the immune responses by stimulation of macrophages.

Cell walls are essential components of viability, morphology, and stress responses. The role of LAMMER kinase in cell wall integrity has been studied in fungi. *An*LkhA is involved in cell wall synthesis by modulating the expression of cell wall synthase genes (*fksA*, *chsC*, and *chsD*) (Choi et al., 2014). The LAMMER kinase *Ca*Kns1 in *C. albicans* is required for cell wall integrity and adherence (Lim et al., 2020b). Although numerous studies have suggested that cell wall biosynthesis is a key factor in pathogenesis, the role of LAMMER kinase in fungal virulence has not been investigated in detail.

Our results provide insights into the role of *Af*LkhA in growth, asexual and sexual development, biofilm formation, alkaline protease activity, GT production, and cell wall PAMP biosynthesis. Here, we describe the involvement of *A. fumigatus* LAMMER kinase in virulence. *Af*LkhA is required for biosynthesis cell wall PAMPs, including β-1,3-glucan and GM. Changes in the PAMPs of fungal cell walls can affect cell wall PAMPs recognition by the PRRs located on the surface of host immune cells. This PRR-PAMP binding stimulates both the innate and adaptive immune systems that are required to fight fungal pathogens.

## Materials and Methods

### Growth Condition and Strains

The *A. fumigatus* strains employed in this study are listed in **Supplementary Table 1**. The supermater strains AFB62 (*MAT1-1*) and AFIR928 (*MAT1-2*) were obtained from the National Institutes of Health (NIH) (Sugui et al., 2011) and maintained in *Aspergillus* minimal medium with glucose (GMM) (Kong et al., 2013).

To construct the disruption cassette, fusion PCR was performed (Szewczyk et al., 2006). Using the genomic DNA of *A. fumigatus* as a template, 5’ and 3’ flanking regions of each *AflkhA* gene were amplified with 5’ UTR *AflkhA* F/R and 3’ UTR *AflkhA* F/R primer set, respectively. The pyrithiamine (PT) resistance gene (*ptrA*) product was amplified with the pTRI plasmid (Takara, Japan) as a template and *ptrA* F/R primer set (Kubodera et al., 2000). The three DNA fragments were then mixed, and fusion PCR was performed with “nested” primers (*AflkhA* nest F/R). The primers used in this study are listed in **Supplementary Table 2**. The disruption cassette was transformed into AFIR928 protoplasts generated using VinoTaste^®^ Pro (Novozymes) [40]. Transformants were selected on GMM with PT, and correct recombination in genomic DNA was confirmed by PCR. To complement Δ*AflkhA*, the predicted promoter region and ORF of the *AflkhA* gene were amplified and transformed into the Δ*AflkhA* strain.

### Phenotypic Analysis

For mycelial production, strains were cultivated in liquid complete medium (YCGMM), which was the GMM supplemented with 0.15% yeast extract and 0.15% casamino acid. To observe conidiophores, conidia were inoculated on solid YCGMM agar blocks and coverslip-cultured for 24 hrs (Lim et al., 2020a). To induce sexual development, the vegetative mass mating (VeM) method was used (Lim and Park, 2019a). Briefly, mycelial balls from isolates of opposite *MAT1-1* and *MAT1-2* mating types were mixed and transferred to oatmeal (Quaker Oats) agar plates, which were sealed with Parafilm and incubated at 30°C in the dark.

For susceptibility testing, conidial suspensions were resuspended to a concentration of 2×10^6^ conidia per mL in distilled water and subjected to 10-fold serial dilution. After dilution, 5 μL of the solution was spotted onto GMM agar and incubated for two days at 37°C (control). Dithiothreitol (DTT) and tunicamycin (Tm), both of which induces UPR, was added to solid GMM to induce endoplasmic reticulum stress. To accomplish ion depletion stress, ethylenediaminetetraacetic acid (EDTA) and ethylene glycol tetraacetic acid (EGTA) were added to the GMM.

### RNA Preparation, cDNA Synthesis, and Quantitative Real-Time PCR

Cells at each developmental stage were ground using liquid nitrogen with a mortar and pestle (Rocha et al., 2015). To extract RNA from cleistothecia, 20 cleistothecia were collected in a screw cap tube, frozen in liquid nitrogen, and vortexed with zirconida/silica beads (Biospec). Total RNA was extracted using TRIzol according to the manufacturer’s protocol (Invitrogen). Briefly, cell powder, 0.5 mm dia. zirconia/silica beads (BioSpec Products), Trizol reagent, and β-mercaptoethanol were added to a 2 mL centrifuge tube and homogenized by bead-beating at 4°C. Additional Trizol reagent and chloroform were added for 10 minutes and the supernatant was collected by centrifugation. RNA was precipitated with 100% ethanol and 8 M LiCl. Dried RNA pellets were dissolved in diethyl pyrocarbonate-treated water at 65°C. Synthesis of cDNA was achieved using 4 µg of extracted RNA, hexamer primers, and M-MLV reverse transcriptase (Enzynomics), as described in the manufacturer’s instructions. RT-qPCR was performed using the Bio-Rad CFX96 Real-Time PCR System (Bio-Rad) and a TOPrealTM qPCR 2X PreMIX Kit (Enzynomics). The transcription levels of target genes were normalized against those of the internal control gene 18S rRNA using the 2^−ΔΔCt^ method as previously described (Park et al., 2015). The primers used for RT-qPCR are listed in **Supplementary Table 2**.

### Microscopy

For microcopy, the Olympus System microscope Model BX51 (Olympus) equipped with UPlanSApo 60X and UPlanFL 100X objective lenses (Olympus) and a stereomicroscope Model SMZ800 (Nikon) were used. Images were captured with a DP71 digital camera (Olympus) and processed using the DP manager imaging software (Olympus).

### GT Analysis

GT production was analyzed in a time-course experiment. Spores (10^5^ conidia/mL) were inoculated in 200 mL of Czapek Dox broth (pH 7.3) and incubated at 37°C. GT was extracted from the supernatant with chloroform, dried, and resuspended in 200 μl of methanol. Approximately 10 μL of each methanol solution and GT standard (Sigma-Aldrich) were loaded onto a TLC silica plate (Silica gel 60 F254; Merck) and developed in a mobile phase composed of chloroform: methanol (90: 10 v/v). The plate was dried at room temperature, and spots of GT were visualized under UV light (254 nm).

### Protease Activity

Spores (5 × 10^3^ conidia) were point inoculated on Czapek Dox medium containing 1% skim milk powder (Difco) instead of sodium nitrate and incubated for three days at 30°C. The degradation halos indicate protease activity. Supernatants were collected from Czapek Dox broth (pH 7.3) containing 1% skim milk powder for three days at 30 ◦C, and then precipitated with four volumes of ice-cold acetone and kept in a cold room overnight. Protein pellets were collected by centrifugation at 5,000 g for 15 minutes at 4°C and air-dried. Protein concentrations were calculated using the BCA Protein Assay Kit (Thermo Fisher Scientific) according to the manufacturer’s protocol. An azocasein assay was also performed as previously described, with some modifications (Shemesh et al., 2017). Azocasein (Sigma-Aldrich) was dissolved at a concentration of 5 mg/mL in an assay buffer containing 50 mM Tris (pH 8.0), 0.2 M NaCl, 5 mM CaCl_2_, and 0.05% Triton X-100. Each protein solution (200 µL) was mixed with 500 µL of azocasein solution and incubated for 90 minutes at 180 rpm at 30 ◦C. The reactions were stopped by adding 200 µL of 12% (v/v) trichloroacetic acid. The reaction mixtures were left at room temperature and centrifuged at 8,000 × g. The supernatant (200 µL) was mixed with 200 µL of 1 M NaOH. The absorbance of the released azo dye was determined at 436 nm.

### Biofilm Formation Assay

Fungal biofilm assays were performed using 12-well plates, as described previously (Lohmar et al., 2019). Wells were inoculated with 1 mL of GMM medium containing 10^5^ conidia and incubated for 16 hrs at 37°C. Biofilms were washed three times with PBS, stained with 1 mL of 0.01% (w/v) crystal violet solution, and incubated for 6 hrs. Destaining was conducted with 1 mL of 30% acetic acid. Adhesion capacity was quantified by measuring absorbance at 550 nm using a spectrophotometer and represented as the absorbance of crystal violet dye bound to the biofilm cells.

### Culture of Macrophages and *A. fumigatus* Stimulation

The mouse alveolar macrophage MH-S cell line (ATCC CRL-2019) was cultured in RPMI-1640 medium (Welgene) supplemented with 0.05 mM 2-mercaptoethanol and 10% fetal bovine serum (FBS), and incubated at 37°C in 5% CO_2_ (Matsunaga et al., 2001).

MAPK phosphorylation was assayed using a previously described method (Dubourdeau et al., 2006). Briefly, MH-S cells were starved for 15–16 hrs in RPMI 1640 medium without FBS, stimulated with 10-fold WT or Δ*AflkhA* conidia, and washed with cold RPMI medium to remove any unbound conidia. After incubation at 37°C in 5% CO_2_ for different incubation times (2, 4, and 6 hrs), the cells were washed with cold DPBS (Welgene) and frozen at -70°C before analysis.

### Phagocytosis Assay

MH-S cells (2 × 10^5^ macrophages/well) were plated on 12-well cell culture plates with cover glass and incubated for 2 hrs at 37°C in 5% CO_2_. The cells were stimulated with WT or Δ*AflkhA* conidia (6 × 10^5^ conidia/well) for 1 hr and washed three times with DPBS. Macrophage conidia were co-cultured for an additional 2 hrs. Wells were then washed with DPBS and stained with calcofluor white (1 μg/mL in DPBS) to label extracellular conidia. The number of *A. fumigatus* conidia phagocytosed by macrophages was quantified as the percentage of macrophages containing at least one ingested conidium. The phagocytic index was calculated as the average number of ingested conidia per phagocytosing macrophage.

### Preparation of Protein Extracts and Immunoblot Analysis

Protein extracts were resuspended in radioimmunoprecipitation assay buffer (Elpis Biotech) with protease inhibitor cocktail (Calbiochem) and phosphatase inhibitor [0.1 M phenylmethylsulfonyl fluoride (PMSF), 0.5 M sodium fluoride, 0.1 M sodium orthovanadate] according to the manufacturer’s protocol. Lysates were collected using a cell scraper and cleared by centrifugation at 13,000 rpm for 15 minutes at 4°C. Equal amounts of total protein extracts were boiled for 5 minutes with 5X sample loading buffer (350 mM Tris-HCl pH 6.8, 600 mM dithiothreitol [DTT], 36% glycerol, 350 mM sodium dodecyl sulfate [SDS], 0.012% [w/v] bromophenol blue), and separated by 10% SDS-PAGE gels. Total proteins were electroblotted onto hybond-P polyvinylidene difluoride (PVDF) membranes (GE Healthcare) and blocked with 5% skim milk in TBST (20 mM Tris-HCl, pH 7.5, 30 mM NaCl, 0.05% Tween 20) for 2 hrs. The membranes were probed with anti-Erk1/2 antibody (1:10000; Cell Signaling Technology), anti-phospho-Erk1/2 antibody (1:10000; Cell Signaling Technology), anti-p38 MAPK antibody (1:10000; BioLegend), anti-phospho-p38 MAPK antibody (1:10000; Santa Cruz Biotechnology), and anti-β-actin antibody (1:10000; Cell Signaling Technology) as primary antibodies, and goat anti-rabbit IgG-HRP (1:10000; Enzo Life Sciences), and goat anti-mouse IgG-HRP (1:5000; Santa Cruz Biotechnology) as secondary antibodies. Immunological detection was performed using an ECL chemiluminescence system (Advansta). Phosphorylation levels were quantified after normalization to the levels of total ERK and p38 using densitometry scanning and ImageJ software.

### Cytokine Measurements

For *in vitro* cytokine expression, co-culture of macrophage cells with *A. fumigatus* conidia was performed as described previously with some modifications (Dubourdeau et al., 2006). Briefly, MH-S cells were added to 96-well plates at a density of 5 × 10^5^ cells. The cells were stimulated with WT or Δ*AflkhA* conidia at a conidium-to-macrophage ratio of 10:1 for 6 hrs and then frozen at -70°C. TNF-α was quantified in supernatants by ELISA using a mouse TNF-α detection kit (Invitrogen, Thermo Fisher Scientific). Data are presented as the average TNF-α value (pg/mL) for biological replicates.

### Zebrafish Infection Assay by Conidial Microinjection

Zebrafish larvae (*foxn1*/*Casper* mutants) at three days post-fertilization (dpf) were obtained from the Zebrafish Center for Disease Modeling (Daejeon, Korea) (Lv et al., 2020). All experiments using zebrafish were conducted with protocols approved by the Animal Ethics Committee of Chungnam National University (CNU-00191). Conidial microinjection was performed as previously described (Bolcome et al., 2008; Knox et al., 2014), but modified to some extent. Conidia from two-day-old cultures at a concentration of 10^8^ conidia/mL were mixed 1:1 with fluorescein isothiocyanate–dextran (Sigma-Aldrich) for clear visualization of injection success and injected into the common cardinal vein/duct of Cuvier. For survival analysis, infected larvae were monitored daily, and mortality was recorded.

### Polysaccharide Analysis

The β-1,3-glucan amounts in conidia were determined using the enzymatic yeast beta-glucan Megazyme kit following the manufacturer’s protocol (Megazyme). Briefly, two-day-old conidia were collected, mixed with 2 M KOH for 30 minutes in an ice water bath, and incubated with 1.2 M sodium acetate buffer (pH 3.8) and Glucazyme™ for 16 hrs at 40°C. After incubation, 10 mL of water was added to each tube, and 100 μL aliquots were transferred to new tubes. GOPOD reagent was added and incubated for 20 minutes at 40°C. The optical density was determined at 510 nm. This test was performed in quadruplicate.

For galactomannan production, 50 mL of modified Brian medium (2% asparagine, 5% glucose, 2.4 g/L NH_4_NO_3_, 10 g/L KH_2_PO_4_, 2 g/L MgSO_4_-7H_2_O, 26 mg/L ZnSO_4_-7H_2_O, 2.6 mg/L CuSO_4_-5H_2_O, 1.3 mg/L Co(NO_3_)_2_- 6H_2_O, 65 mg/L CaCl_2_, pH 5.4) was inoculated with 5 × 10^7^ conidia and incubated at 37°C at 180 rpm for 24 hrs (Brian et al., 1961; Fontaine et al., 2011; Gravelat et al., 2013). Culture supernatants were prepared by filtration of the culture on Miracloth. Extracellular GM content in the culture supernatant was assayed by ELISA using the GM Ag kit (San Diego, USA), following the manufacturer’s instructions.

## Data Availability Statement

Publicly available datasets were analyzed in this study. This data can be found here: A FungiDB (https://fungidb.org/fungidb/. The original contributions presented in the study are included in the article/**Supplementary Material**. Further inquiries can be directed to the corresponding author.

## Ethics Statement

The animal study was reviewed and approved by The Animal Ethics Committee of Chungnam National University.

## Author Contributions

J-YL, C-HK, and H-MP designed the experiments. J-YL, YK, SW, JJ, and Y-RL performed the experiments. J-YL, YK, SW, and JJ performed interpretation of the results and data analysis. C-HK and H-MP contributed resource. J-YL and H-MP wrote the manuscript. All authors contributed to the article and approved the submitted version.

## Funding

This research was supported by the grants from the National Research Foundation of Korea (2020R1F1A1073075 to H-MP, 2020R1A6A3A01099306 to J-YL, and 2018M3A9B8021980 to C-HK).

## Conflict of Interest

The authors declare that the research was conducted in the absence of any commercial or financial relationships that could be construed as a potential conflict of interest.

## Publisher’s Note

All claims expressed in this article are solely those of the authors and do not necessarily represent those of their affiliated organizations, or those of the publisher, the editors and the reviewers. Any product that may be evaluated in this article, or claim that may be made by its manufacturer, is not guaranteed or endorsed by the publisher.
