# Corrigendum: The LAMMER Kinase, LkhA, Affects *Aspergillus fumigatus* Pathogenicity by Modulating Reproduction and Biosynthesis of Cell Wall PAMPs

**DOI:** 10.3389/fcimb.2021.797626

**Published:** 2022-01-19

**Authors:** Joo-Yeon Lim, Yeon Ju Kim, Seul Ah Woo, Jae Wan Jeong, Yu-Ri Lee, Cheol-Hee Kim, Hee-Moon Park

**Affiliations:** ^1^ Laboratory of Cellular Differentiation, Department of Microbiology and Molecular Biology, College of Bioscience and Biotechnology, Chungnam National University, Daejeon, South Korea; ^2^ Institute of Biotechnology, Chungnam National University, Daejeon, South Korea; ^3^ Laboratory of Developmental Genetics, Department of Biology, College of Bioscience and Biotechnology, Chungnam National University, Daejeon, South Korea

**Keywords:** *Aspergillus fumigatus*, gene regulation, fungal development, interactions with host cells, molecular mechanisms of fungal pathogenesis, pathogen associated molecular patterns (PAMPs)

In the original article, there was a mistake in ** **Figure 5A** ** as published. **DAPI should be changed to CFW in **Figure 5A** **. The corrected ** **Figure 5A** ** appears below.

**Figure 5:**
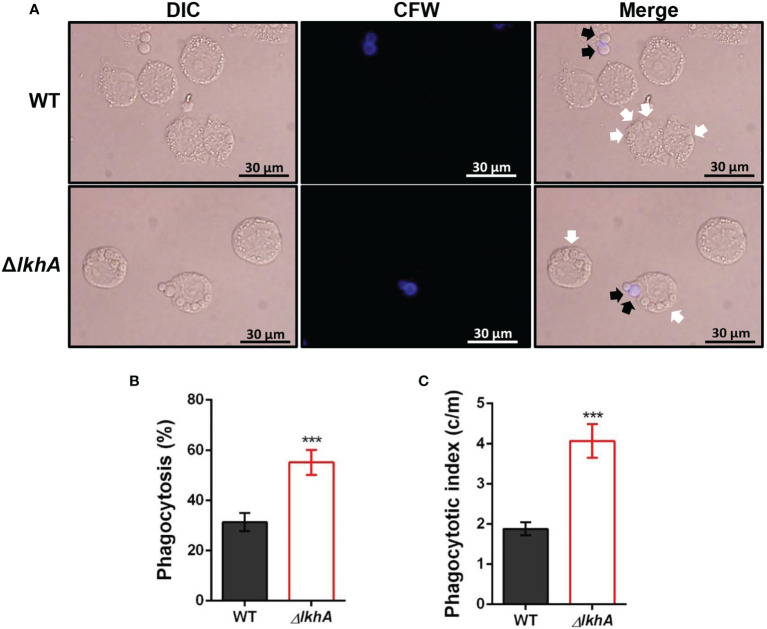
Alveolar macrophage response to *ΔAflkhA* conidia. MH-S murine alveolar macrophages were challenged with a three-fold concentration of WT and *ΔAflkhA* conidia and then incubated for 4 hrs at 37°C in an atmosphere of 5% CO2. **(A)** Microscopic analysis of the uptake of the fungal conidia by MH-S macrophages. External conidia (black arrows) were stained by calcofluor white (1 mg/mL in DPBS). White arrows indicate conidia endocytosed by macrophage cells. **(B)** Phagocytosis of conidia. The percentage of macrophages containing more than one ingested conidia was counted (n = 18). **(C)** Phagocytosis index. The average number of indigested conidia per macrophage (c/m) was calculated (n = 40). Statistical analysis was performed using the student’s t-test. ***P < 0.001.

The authors apologize for this error and state that this does not change the scientific conclusions of the article in any way. The original article has been updated.

## Publisher’s Note

All claims expressed in this article are solely those of the authors and do not necessarily represent those of their affiliated organizations, or those of the publisher, the editors and the reviewers. Any product that may be evaluated in this article, or claim that may be made by its manufacturer, is not guaranteed or endorsed by the publisher.

