# Supplementary material for: The LAMMER Kinase, LkhA, Affects Aspergillus fumigatus Pathogenicity by Modulating Reproduction and Biosynthesis of Cell Wall PAMPs
Source: Front Cell Infect Microbiol. 2021 Oct 13;11:756206. doi: 10.3389/fcimb.2021.756206 (PMC8548842; doi:10.3389/fcimb.2021.756206)
Supplement: Supplementary file 1 [file DataSheet_1.pdf]

Supplementary Material

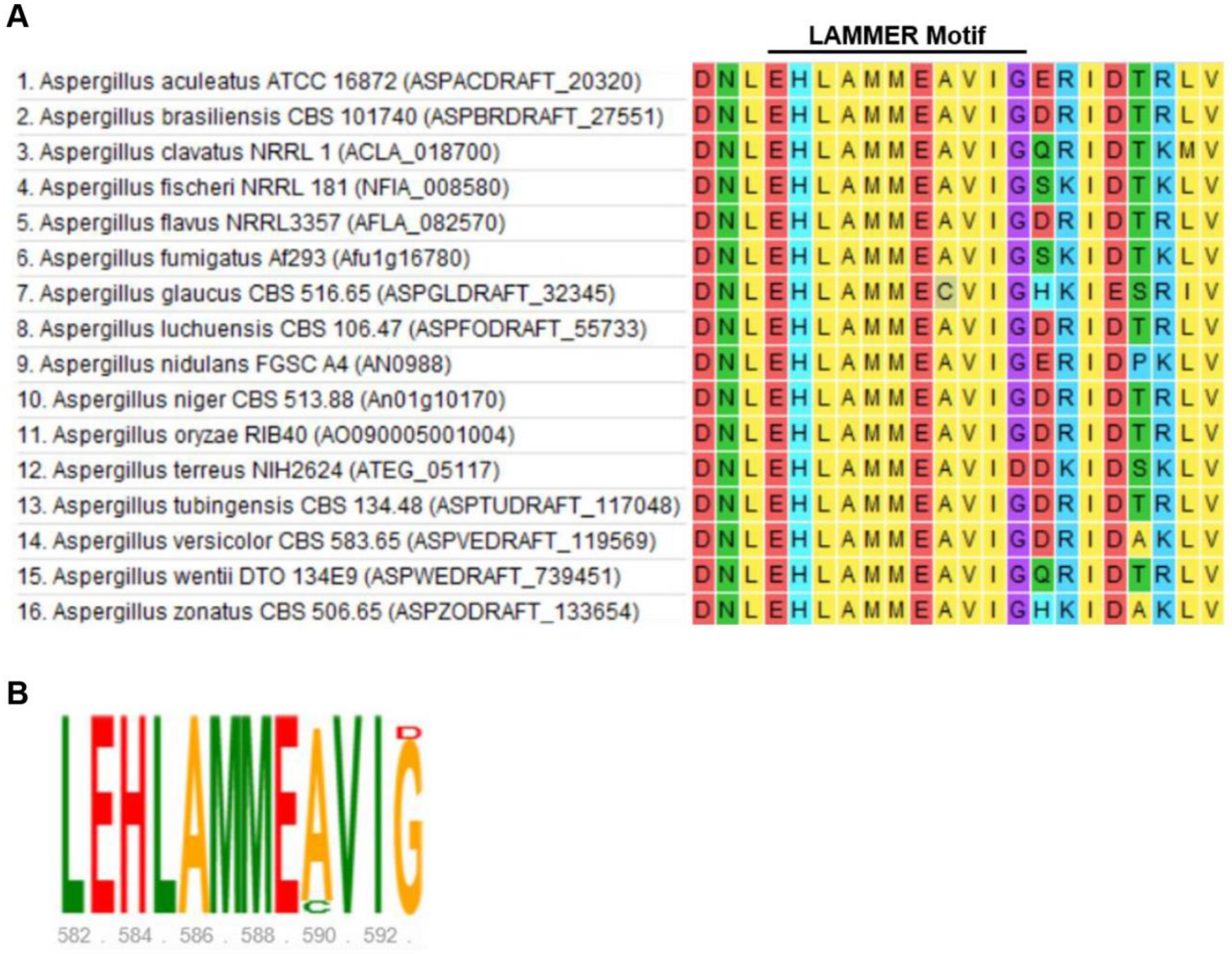

Supplementary Figure 1. LAMMER motif analysis in *Aspergillus* spp. (A) Multiple alignment of amino acid sequences in the LAMMER kinase orthologs in 16 *Aspergillus* species. A FungiDB (https://fungidb.org/fungidb/) and MEGA7 were used to show homology of amino acid sequences and align sequences. (B) Sequence logos for LAMMER motif in *Aspergillus* species. Sequence logos were generated with the online tool MPI Bioinformatics toolkit (https://toolkit.tuebingen.mpg.de/tools/alnviz).

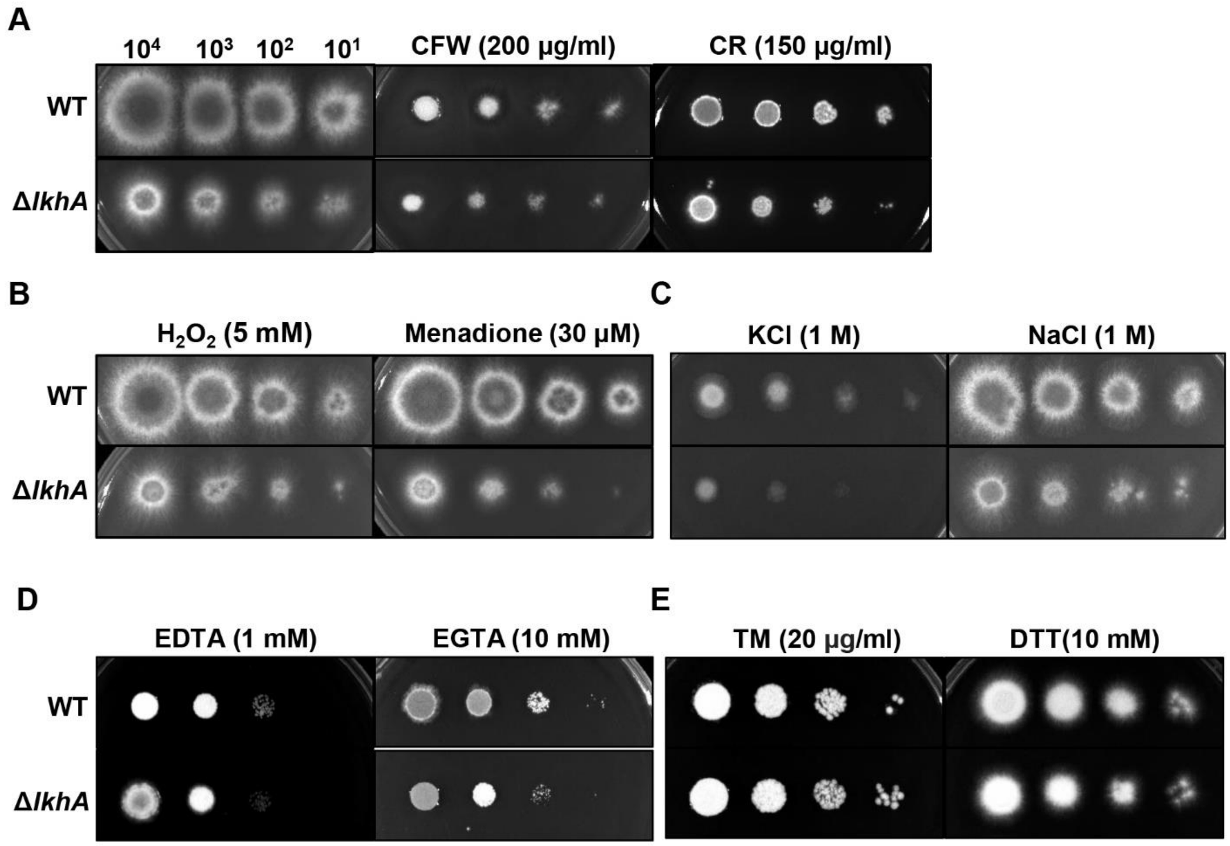

Supplementary Figure 2. Sensitivity test for various stress. Spores with 10-fold serial dilutions (10^4, 10^3, 10^2, and 10^1) were spotted onto the GMM containing the stress-imposing agents. (A) Sensitivity tests for cell wall damage stress CFW and CR. CFW: calcofluor white, CR: congo red (B) Sensitivity test for oxidative stress (H_2O_2 and menadione). (C) Sensitivity test for osmotic stress (KCl and NaCl). (D) Sensitivity test for ion depletion stress (EDTA and EGTA). (E) Sensitivity test for endoplasmic reticulum stress (TM and DTT). TM: tunicamycin, DTT: dithiotreitol.

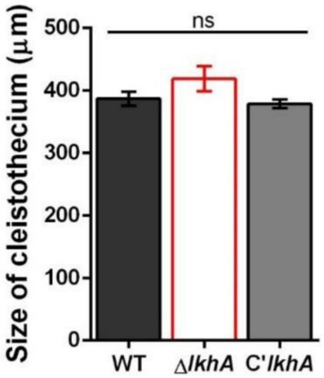

Supplementary Figure 3. Size of cleistothecium. The diameter of randomly selected cleistothecia were measured using the ImageJ software. Not significantly different is indicated by "ns".

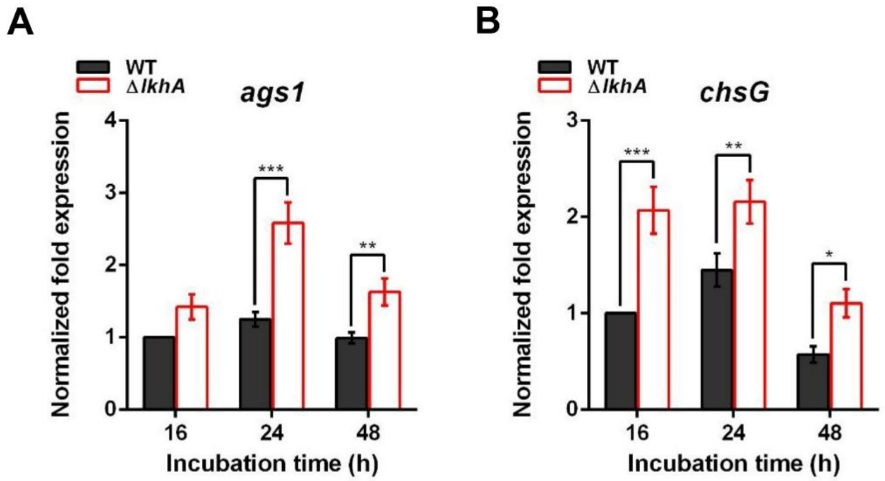

Supplementary Figure 4. Expression levels of the α-1,3-glucan synthase and chitin synthase genes, *ags1* and *chsG*, respectively. Spores were inoculated in MM liquid culture and incubated for the indicated time periods. Total RNA was extracted, and RT-qPCR analysis was performed using the 18S rRNA gene as an internal control. * P<0.05, ** P<0.01, *** P<0.01.

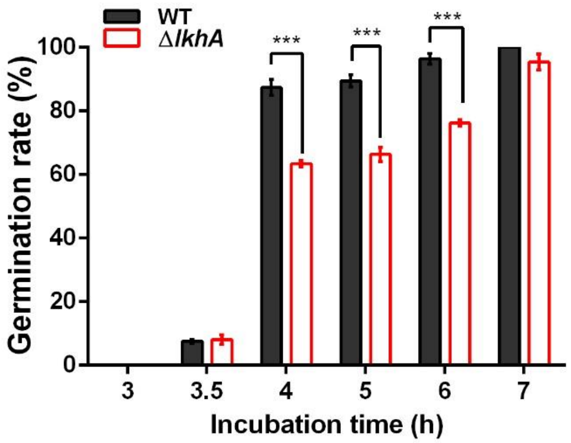

Supplementary Figure 5. Germination rate of conidia. Kinetics of germ tube formation in inoculated WT and Δ*AflkhA* conidia were investigated in YCGMM broth at 37℃. The number of germinating conidia was presented as a percentage of the total number of conidia. *** P<0.01.
